# Cool head-out water immersion does not alter cerebrovascular reactivity to hypercapnia despite elevated middle cerebral artery blood velocity: A pilot study

**DOI:** 10.1371/journal.pone.0298587

**Published:** 2024-03-13

**Authors:** Morgan L. Worley, Emma L. Reed, Nathan Klaes, Zachary J. Schlader, Blair D. Johnson

**Affiliations:** 1 Center for Research and Education in Special Environments, Department of Exercise and Nutrition Sciences, School of Public Health and Health Professions, University at Buffalo, Buffalo, NY, United States of America; 2 Department of Human Physiology, College of Arts and Sciences, University of Oregon, Eugene, OR, United States of America; 3 Department of Kinesiology, School of Public Health-Bloomington, Indiana University, Bloomington, IN, United States of America; Academic Teaching Hospital of the University of Goettingen / University of Alexandria, GERMANY

## Abstract

Episodic increases in cerebral blood flow (CBF) are thought to contribute to improved cerebrovascular function and health. Head-out water immersion (HOWI) may be a useful modality to increase CBF secondary to the hydrostatic pressure placed on the body. However, it is unclear whether water temperatures common to the general public elicit similar cerebrovascular responses. We tested the hypothesis that mean middle cerebral artery blood velocity (MCAv_mean_) and cerebrovascular reactivity to CO_2_ (CVR_CO2_) would be higher during an acute bout of thermoneutral (TN; 35°C) vs. cool (COOL; 25°C) HOWI. Ten healthy participants (age: 23±3 y; 4 women) completed two randomized HOWI visits. Right MCAv_mean_, end-tidal CO_2_ (PETCO_2_) mean arterial pressure (MAP), and MCA conductance (MCAv_mean_/MAP) were continuously recorded. CVR_CO2_ was assessed using a stepped hypercapnia protocol before (PRE), at 30 minutes of HOWI (HOWI), immediately after HOWI (POST-1), and 45 minutes after HOWI (POST-2). Absolute values are reported as mean ± SD. MCAv_mean_, PETCO_2_, MAP, and CVR_CO2_ were not different between conditions at any timepoint (all *P*≥0.17). In COOL, MCAv_mean_ increased from PRE (61±9 cm/s) during HOWI (68±11 cm/s), at POST-1 (69±11 cm/s), and POST-2 (72±8 cm/s) (all P<0.01), and in TN from PRE to POST-1 (66±13 vs. 71±14 cm/s; P = 0.05). PETCO_2_ did not change over time in either condition. In COOL, MAP increased from PRE (85±5 mmHg) during HOWI (101±4 mmHg), at POST-1 (97±7 mmHg), and POST-2 (96±9 mmHg), and in TN from PRE (88±5 mmHg) at HOWI (98±7 mmHg) and POST-1 (99±8 mmHg) (all P<0.01). In COOL, CVR_CO2_ increased from PRE to HOWI (1.66±0.55 vs. 1.92±0.52 cm/s/mmHg; P = 0.04). MCA conductance was not different between or within conditions. These data indicate that 30 minutes of cool HOWI augments MCAv_mean_ and that the increase in MCAv_mean_ persists beyond cool HOWI. However, cool HOWI does not alter CVR_CO2_ in healthy young adults.

## Introduction

Thermoneutral head-out water immersion has garnered attention as a potential modality to induce elevations in cerebral blood flow (CBF). It is speculated that episodic increases in CBF and potentially shear stress, which augments endothelial-derived nitric oxide bioavailability [1] and brain-derived neurotrophic factor [2–4], may contribute to improved cerebrovascular health [5, 6]. During thermoneutral water immersion, CBF increases via immersion-induced elevations in arterial CO_2_ and blood pressure secondary to the hydrostatic pressure placed on the body [7–10]. Alterations in these two primary modulators of CBF (i.e., arterial CO_2_ and blood pressure) are influenced by factors such as water temperature and the duration of immersion. To date, the majority of acute water immersion studies have utilized water temperatures (30–35°C) that are close to thermoneutral. These conditions elicit increases in PETCO_2_ [8–10] and mean arterial pressure [8, 10], which promotes increases in middle cerebral artery (MCA) [8–10] and posterior cerebral artery blood velocities [8], but no change in common carotid artery (CCA) blood flow [8] from 1–30 minutes of head-out immersion. Although the increase in intracranial artery blood velocity may be beneficial as a repetitive stimulus, a 30-35°C body of water may not be readily available to the general population. For instance, US Master’s Swimming and the World Health Organization have determined that a standard lap swimming pool should be 25–28°C for safety and comfort [11]. Thus, due to its accessibility and general use by various populations, it is important to determine if this water temperature alters cerebrovascular hemodynamics differently than the standard thermoneutral water (~30–35°C) used in laboratory studies [8–10, 12]. Since these slightly cooler water temperatures are below average skin temperature, it is possible that intracranial artery blood velocity would be reduced if hyperventilation-induced hypocapnia [13] is present.

Currently, no studies have investigated the acute effects of cool HOWI on intracranial artery blood velocity and cerebrovascular reactivity to CO_2_, which is an important marker of microvascular brain health and disease risk [14]. Cerebrovascular reactivity to CO_2_ assesses the ability of the blood vessels in the brain to constrict or dilate in response to a vasoactive stimulus, such as changes in arterial CO_2_. We have previously demonstrated that 30 minutes of thermoneutral water immersion does not alter cerebrovascular reactivity to CO_2_ during a fixed inspired stepped hypercapnia challenge [10]; however, it was reduced during 1 hour of thermoneutral water immersion using a rebreathe technique [9]. Therefore, it remains unclear if water immersion alters cerebrovascular reactivity to CO_2_, and whether this component of cerebrovascular function is effected by water temperature. We speculate that if cool water immersion induces hyperventilation, hypocapnia will ensue and decrease MCA blood velocity. Local vasoconstriction in the MCA then may impair the MCA ability to vasodilate in response to hypercapnia. Therefore, the purpose of our study was to determine if 30 minutes of HOWI in cool water alters MCA blood velocity and cerebrovascular reactivity to CO_2_ during and following immersion. We tested the hypotheses that, compared to thermoneutral HOWI, cool HOWI: 1) decreases MCA blood velocity and 2) reduces cerebrovascular reactivity to CO_2_ in healthy young adults. The findings from this pilot study could be utilized to inform future immersion-based interventions for cerebrovascular health.

## Methods

### Participants

An *a priori* power calculation was not completed for this pilot study since our main outcome measures had yet to be reported using similar cool water conditions. Thus, ten healthy adults (age: 23 ± 3 y; height: 173 ± 9 cm; weight: 69 ± 12 kg; BMI: 23 ± 3 kg/m^2^; 4 women) completed this pilot study that consisted of three visits: one informed consent/screening visit and two experimental study visits. All participants were fully informed of the experimental procedures prior to providing informed, written consent. The study was approved by the Institutional Review Board at the University at Buffalo and was performed in accordance with the standards set by the latest revision of the Declaration of Helsinki, except for registration in a database. The study was completed between March 4^th^, 2019 and May 8^th^, 2020. Participants were excluded for self-reporting tobacco use; beta-blocker, alpha-blocker, angiotensin converting enzyme inhibitor, angiotensin receptor blocker use, or the use of any other medication that affects blood pressure or the autonomic nervous system; current or history of digestive tract disorders (irritable bowel syndrome, hemorrhoids, diarrhea, constipation, cancer, surgery); pre-existing autonomic, cardiovascular, metabolic, respiratory, or endocrine disorder; major depression; pregnancy; or breastfeeding. Three women self-reported not using contraceptives and one woman reported using an intrauterine device (Skyla).

### Instrumentation and measurements

Height and nude body weight were measured with a stadiometer and scale (Sartorius, Bohemia, NY). Urine specific gravity was measured via refractometry (Atago USA, Bellevue, WA) before each experimental visit. An intestinal temperature telemetry pill (HQ Inc., Palmetto, FL, USA) was ingested ~1 hour prior to baseline measurements [15] and intestinal temperature was recorded every 5 minutes throughout experimental visits. Heart rate was continuously measured using a 3-lead electrocardiogram (ECG; DA100C, Biopac Systems, Goleta, CA). Blood pressure was continuously measured using finger photoplethysmography on the right hand that was supported out of the water (Finometer Pro, FMS, Amsterdam, Netherlands). Blood pressure was corrected to the heart level using a height correction sensor. End-tidal CO_2_ tension (PETCO_2_) was continuously measured through a mouthpiece (with nose clip) using capnography (Nonin Medical, Inc., Plymouth, MN) providing an index of the partial pressure of arterial carbon dioxide [16]. Right MCA mean blood velocity (MCAv_mean_) was continuously measured as an index of blood flow to the anterior cerebral circulation via a 2 MHz transcranial Doppler probe (DWL USA, Inc., Germany, Europe) using insonation techniques described in detail by Willie *et al*. [17]. We chose the MCA as our intracranial vessel of interest since it is the largest of the cerebral arteries and is the most affected by cerebrovascular insults (e.g., stroke) [18, 19]. The depth, gain, and location of the right MCA were recorded during the first experimental visit and used in the second experimental visit. B-mode and pulse wave velocity images of the right common carotid artery (CCA) were taken prior to each cerebrovascular reactivity test using a 10 MHz linear transducer (Vivid 7 Dimension, GE, 2010). We chose the CCA as an extracranial vessel of interest as changes within this major artery, such as diameter, are an indicator of cardiovascular and cerebrovascular health [20–22]. The sample volume was placed in the middle of the vessel and covered approximately 2/3 of the diameter. The insonation angle was always set to <60°. Both the sample volume and insonation angle were maintained within and between visits. All CCA images were obtained by the same sonographer (MLW). The within-subject test-retest coefficient variation for CCA diameter was 1.2±1.0% and CCA blood flow was 3.2±2.8% (*n* = 8).

### Experimental approach

Participants completed two randomized experimental visits that consisted of thermoneutral water immersion (TN; 35°C water) and cool water immersion (COOL; 25°C water) for 45 minutes each. We chose our cool water immersion temperature to reflect the temperature of a typical lap swimming pool. Experimental visits were completed at least 7 days apart and at the same time of day (±1 hour). Women were tested during the first 10 days of menstruation (thermoneutral visit: 6±3 days; COOL visit: 7±4 days). Participants were instructed to report to the laboratory after abstaining from exercise, alcohol, caffeine and other stimulants for 12 hours, and food for 2 hours. Upon arrival, participants ingested a temperature telemetry pill and provided a urine sample and nude body weight in a private room. At this point, participants were not allowed to consume fluids for the remainder of the experimental visit. Urine samples were assessed for hydration status via urine specific gravity and confirmation of a negative pregnancy status (women only) via a urine pregnancy test. A urine specific gravity of ≤1.025 was used as a study visit inclusion criteria and no participants exceeded a urine specific gravity of 1.025 prior to testing. After a urine sample and nude body weight were obtained, participants entered the empty water immersion tank and assumed a seated position with their right arm supported on a platform near heart level. Participants were then instrumented with equipment to continuously measure heart rate, blood pressure, PETCO_2_, and right MCAv_mean_. After >10 minutes of quiet rest, pre-immersion (PRE) data collection was initiated which included 5-minutes of baseline data collection followed by a cerebrovascular reactivity test and a 5-minute recovery period. Following PRE, the immersion tank was rapidly filled with water to the level of the mid-sternal body (~4 minutes). Cerebrovascular reactivity testing was initiated at 30 minutes of HOWI (HOWI) resulting in participants being in the water for a total of 45 minutes. We chose to test at 30 minutes of HOWI based on our prior work that showed an increase in MCAv_mean_ in thermoneutral water (35°C) [10]. Furthermore, Bailey *et al*. [23] investigated how 30 minutes of hot water immersion performed 3 times per week for 8 weeks influenced MCAv_mean_. Thus, we aimed to understand the acute effects of 30 minutes of a cooler water temperature on middle cerebral artery blood velocity and cerebrovascular reactivity to CO_2_ (CVR_CO2_). The water immersion tank was then rapidly emptied (~4 minutes) after the cerebrovascular reactivity test was completed. Once the water was below the participant’s feet, a 45-minute dry recovery period commenced. Participants were allowed to towel dry and wrap their upper/lower body in a towel while the tank was being emptied (i.e., before the start of POST-1) and/or between post-testing timepoints. Baseline data and cerebrovascular reactivity tests were conducted immediately post-HOWI (POST-1) and 45 minutes post-HOWI (POST-2). We chose to test after HOWI to determine if a reduction in body temperature following immersion impacted cerebrovascular hemodynamics as well as determine if any changes that occurred during immersion persisted or if any potentially beneficial alterations emerged in the post-immersion state.

We assessed CVR_CO2_ in the right MCA using a fixed inspired hypercapnia protocol [24–26]. Participants inhaled 3%, 5%, and 7% CO_2_ for 3 minutes each in a step-wise fashion through a five-way switching valve (Hans Rudolph, Inc., Shawnee, KS, USA). All of the hypercapnic gases contained 21% oxygen and were balanced with nitrogen. We chose to utilize a hypercapnic protocol as this component of CVR_CO2_ appears to be altered following a mild traumatic brain injury [27–31] and has demonstrated to be predictive of stroke in patients with carotid artery disease [32]. Thus, data from our pilot study could be useful for the design of a water immersion intervention aimed at improving intracranial artery blood velocity and cerebrovascular function.

### Data acquisition

Heart rate (1000 Hz), blood pressure (62.5 Hz), stroke volume (62.5 Hz), PETCO_2_ (62.5 Hz), respiratory rate (62.5 Hz), and MCAv_mean_ (1,000 Hz) were continuously recorded using a data acquisition system (Biopac MP150, AcqKnowledge, 4.2.0, Goleta, CA). Stroke volume was estimated using Modelflow (*n* = 9 due to signal loss) [33]. Cardiac output was calculated as the product of heart rate and stroke volume (*n* = 9). Total peripheral resistance was calculated as the quotient of mean arterial pressure and cardiac output (*n* = 9). MCA conductance was calculated as the quotient of MCAv_mean_ and mean arterial pressure (*n* = 10). Please refer to the figure legends for data points at each timepoint.

### Data analysis

#### Cerebrovascular reactivity to CO_2_ (CVR_CO2_)

Absolute mean values were calculated over the final two minutes of baseline and every 30 seconds throughout the 9-minute stepped hypercapnia protocol. Absolute cerebrovascular reactivity was calculated as the slope of the linear regression line between MCAv_mean_ and PETCO_2_ (CVR_CO2-VELOCITY_) and between MCA conductance and PETCO_2_ (CVR_CO2-CONDUCTANCE_). Additionally, the change in PETCO_2_ and mean arterial pressure were calculated from baseline to the last 30 s of 7% CO_2_ inhalation to provide insight on if cerebral artery blood velocity modulators differed across testing timepoints.

#### Common carotid artery ultrasound

We obtained CCA images from 7 of 10 participants. Offline calculations for CCA mean blood velocity (cm/s) and diameter (cm) values were obtained from longitudinal sections in brightness mode (B-mode) and pulse-wave mode (PW-mode). We then used the mean values to calculate blood flow (ml/min) and shear rate (s^-1^) [34]. Blood flow was calculated as:

$$Blood\;flow=\left\{Mean\;blood\;velocity∙\pi ∙{\left[\frac{Diameter}{2}\right]}^{2}∙60\right\}$$

Shear rate was calculated as:

$$Shear\;Rate=4∙\frac{Mean\;blood\;velocity}{Diameter}$$

### Statistical analysis

Data was analyzed using PRISM software (Version 8, GraphPad Software, La Jolla, CA) using the absolute values. Normal distributions for all variables were confirmed via Shapiro-Wilk normality tests prior to running statistical analyses. We used a two-tailed paired t-test to determine if PRE urine specific gravity was different between experimental visits. Linear mixed-effects models were used to compare absolute values within and between experimental visits. If a mixed-effects model revealed a significant interaction or main effect [35, 36], we used Bonferroni post hoc analyses to determine where differences occurred. Cohen’s *d* effect sizes were calculated for absolute values between experimental visits [37]. Cohen’s *d* effect sizes were interpreted as small (*d* = 0.2), medium (*d* = 0.5), and large (*d* = 0.8) [38, 39]. Negative Cohen’s d values were interpreted as small (*d* = -0.2), medium (*d* = -0.5) and large (*d* = -0.8) and left as negatives as this provides insight on the direction of effect. Exact *P*-values are reported where appropriate. Statistical significance was set *a priori* at P≤0.05. Data are reported as the change from PRE (mean ± SD).

## Results

### Hydration status and intestinal temperature

**Fig 1:**
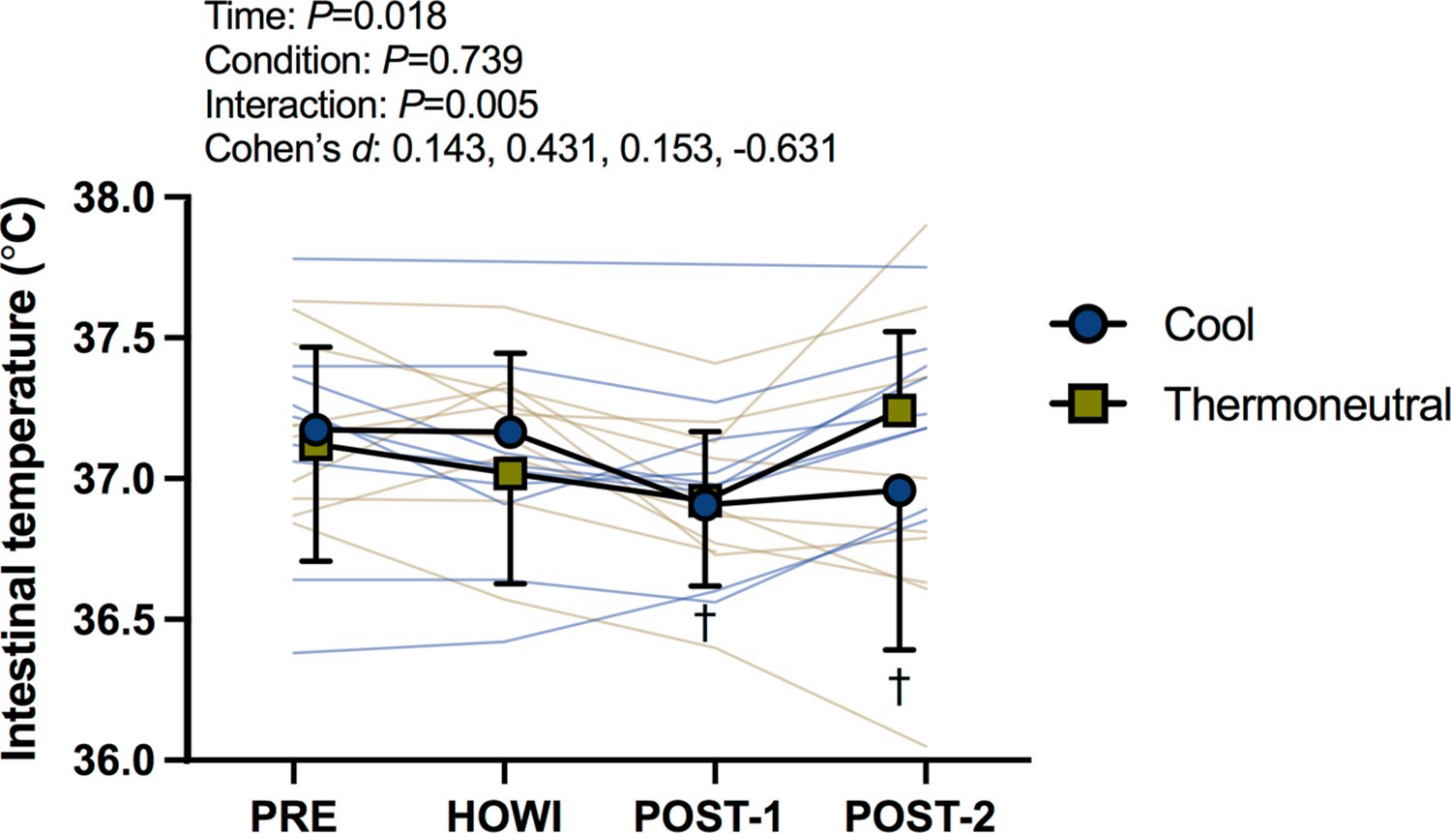
Intestinal temperature. Intestinal temperature at pre-immersion (PRE), 30 minutes of head-out water immersion (HOWI), immediately post-immersion (POST-1) and 45 minutes post-immersion (POST-2) in thermoneutral (35*°*C) and cool water (25*°*C). A linear mixed-effects model was used to compare the absolute mean values. *Post hoc* tests using the Bonferroni’s correction for multiple comparisons were used where appropriate. † = different than PRE. n = 10 at PRE and HOWI. n = 9 at POST-1 and POST-2.

Urine specific gravity and intestinal temperature were not different between conditions at PRE (**Table 1**). In TN, intestinal temperature did not differ from PRE (all *P*≥0.427). In COOL, intestinal temperature was lower than PRE at POST-1 (*P* = 0.005) and POST-2 (*P* = 0.018). Intestinal temperature was not different between conditions (all *P*≥0.081) (**Fig 1**).

**Table 1:** Pre-immersion baseline values.

Variable	Thermoneutral	Cool	P-value	n
**Urine specific gravity**	1.004 ± 0.007	1.012 ± 0.009	0.693	10
**Intestinal temperature (°C)**	37.2 ± 0.4	37.2 ± 0.3	0.999	10
**Heart rate (bpm)**	79 ± 18	77 ± 13	0.999	10
**Stroke volume (mL)**	87 ± 22	81 ± 18	0.344	9
**Cardiac output (L/min)**	6.6 ± 2.2	6.0 ± 1.4	0.333	9
**Mean arterial pressure (mmHg)**	88 ± 5	85 ± 5	0.999	10
**Total peripheral resistance (mmHg/L/min)**	14.5 ± 3.6	15.0 ± 2.9	0.999	9
**Respiratory rate (breaths/min)**	15 ± 3	15 ± 3	0.999	10
**PETCO**_**2**_ **(mmHg)**	44 ± 4	43 ± 3	0.999	10
**MCAv**_**mean**_ **(cm/s)**	66 ± 13	61 ± 9	0.320	10
**MCA conductance (cm/s/mmHg)**	0.76 ± 0.16	0.73 ± 0.14	0.999	10
**CVR**_**CO2**_ **(cm/s/mmHg)**	1.93 ± 0.50	1.61 ± 0.55	0.173	10
**CVR**_**CO2-CONDUCTANCE**_ **(cm/s/mmHg/mmHg PETCO**_**2**_**)**	0.01 ± 0.01	0.01 ± 0.00	0.999	10
**CCA diameter (cm)**	0.59 ± 0.06	0.53 ± 0.08	0.050	7
**CCA mean blood velocity (cm/s)**	44 ± 10	42 ± 5	0.999	7
**CCA blood flow (ml/min)**	709 ± 107	564 ± 152	0.174	7
**CCA shear rate (s** ^**-1**^ **)**	306 ± 86	325 ± 67	0.999	7

PETCO_2_ = partial pressure of end-tidal carbon dioxide; MCA = middle cerebral artery; MCAv_mean_ = middle cerebral artery mean blood velocity; CVR_CO2_ = cerebrovascular reactivity to hypercapnia; CCA = common carotid artery. A two-tailed paired t-test is reported for urine specific gravity. Mixed-effects model are presented for all other variables. Statistical significance set at P≤0.05.

### Cardiovascular responses

**Fig 2:**
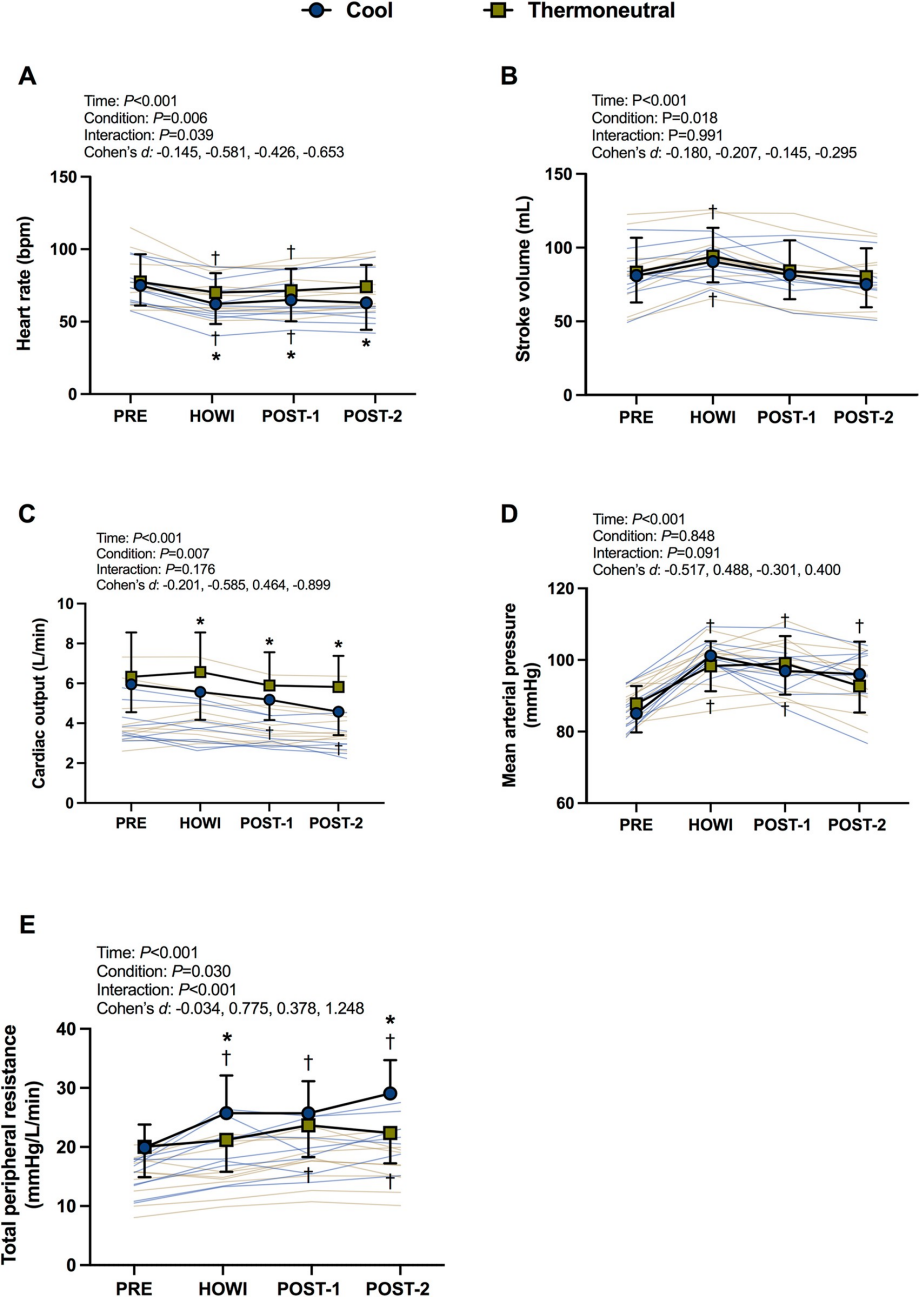
Cardiovascular responses. Heart rate (A), stroke volume (B), cardiac output (C), mean arterial pressure (D), and total peripheral resitance (E) at pre-immersion (PRE), 30 minutes of head-out water immersion (HOWI), immediately post-immersion (POST-1) and 45 minutes post-immersion (POST-2) in thermoneutral (35*°*C) and cool water (25*°*C). A linear mixed-effects model was used to compare the absolute mean values. *Post hoc* tests using the Bonferroni’s correction for multiple comparisons were used where appropriate. † = different than PRE. * = different between conditions. n = 10. Cohen’s *d* values are presented as PRE, HOWI, POST-1, and POST-2. Heart rate and mean arterial pressure: n = 10 at PRE, HOWI and POST-1, and n = 8 at POST-2. Stroke volume, cardiac output, and total peripheral resistance: n = 9 at PRE, HOWI, and POST-1, and n = 7 at POST-2.

Cardiovascular variables were not different between conditions at PRE (**Table 1**). In TN, heart rate was lower than PRE at HOWI (*P*<0.001) and POST-1 (*P* = 0.004). In COOL, heart rate was lower than PRE at every time point (all *P*<0.001). Heart rate was higher in TN vs. COOL during HOWI through POST-2 (all *P*<0.041) with medium effect sizes at HOWI (*d* = -0.581) and POST-2 (*d* = -0.653) (**Fig 2A**). In TN, stroke volume was greater than PRE at HOWI (*P* = 0.002). In COOL, stroke volume was greater than PRE at HOWI (*P* = 0.010). Stroke volume was not different between conditions (all *P* ≥0.160) (**Fig 2B**). In TN, cardiac output did not differ from PRE (all *P≥*0.082). In COOL, cardiac output was lower than PRE at POST-1 (*P*≤0.008) and POST-2 (*P*<0.001). Cardiac output was higher in TN vs. COOL during HOWI (*P* = 0.003, *d* = -0.585), at POST-1 (*P* = 0.038, *d* = 0.464), and at POST-2 (*P* = 0.010, *d* = -0.899) (**Fig 2C**). In TN, mean arterial pressure was greater than PRE during HOWI (*P*<0.001) and at POST-1 (*P*<0.001), and was nearly significant at POST-2 (*P* = 0.062). In COOL, mean arterial pressure was greater than PRE at every time point (all *P*<0.001). Mean arterial pressure was not different between conditions at any timepoint (all *P*≥0.864) (**Fig 2D**). In TN, total peripheral resistance was greater than PRE at POST-1 (*P*<0.001) and POST-2 (*P* = 0.039). In COOL, total peripheral resistance was greater than PRE at every time point (all *P*<0.001). Total peripheral resistance was greater in COOL vs. TN during HOWI (*P* = 0.011, d = 0.775) and at POST-2 (*P* = 0.002, d = 1.248) (**Fig 2E**).

### Respiratory rate and PETCO_2_

**Fig 3:**
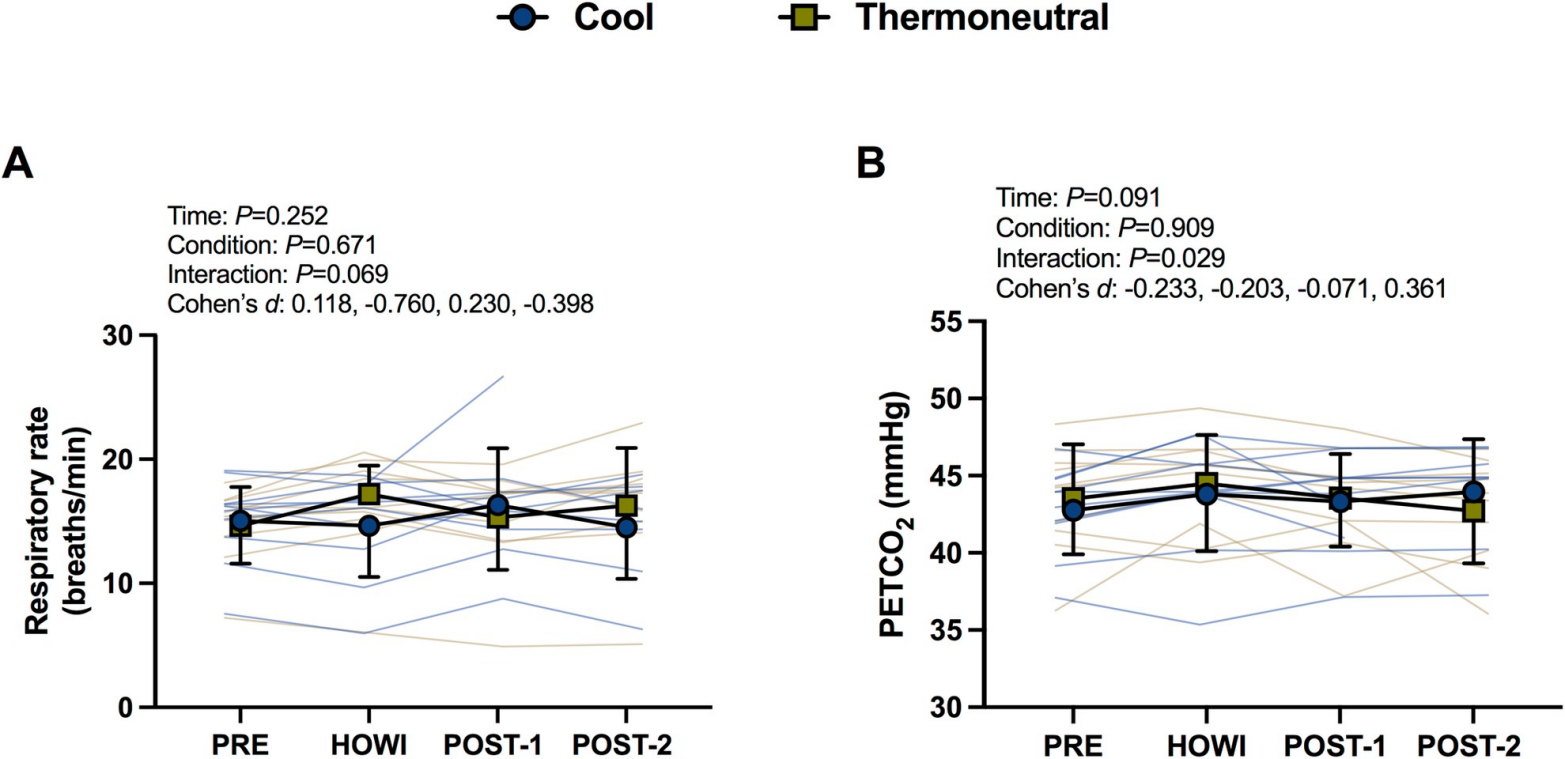
Respiratory rate (A) and end-tidal CO_2_ tension (B) at pre-immersion (PRE), 30 minutes of head-out water immersion (HOWI), immediately post-immersion (POST-1) and 45 minutes post-immersion (POST-2) in thermoneutral (35*°*C) and cool water (25*°*C). Linear mixed-effects models were used to compare the absolute mean values. *Post hoc* tests using the Bonferroni’s correction for multiple comparisons were used where appropriate. Cohen’s *d* values are presented as PRE, HOWI, POST-1, and POST-2. Respiratory rate: n = 10 at PRE, n = 9 at HOWI, n = 10 at POST-2, and n = 8 at POST-2. PETCO_2_: n = 10 at PRE, HOWI, and POST-1, and n = 8 at POST-2.

Baseline respiratory rate and PETCO_2_ were not different between conditions at PRE (**Table 1**). There was no significant main effect or interaction for respiratory rate (*P*≥0.069). However, large effect sizes were observed between conditions during HOWI (*d* = -0.760) (**Fig 3A**). The mixed-effects model revealed a significant interaction for PETCO_2_ (*P* = 0.029), but multiple comparisons did not reveal where differences occurred. In COOL, PETCO_2_ did not differ over time (all *P*≥0.079). PETCO_2_ was not different between conditions (all *P*≥0.469) (**Fig 3B**).

### Cerebrovascular responses

#### Baseline responses

**Fig 4:**
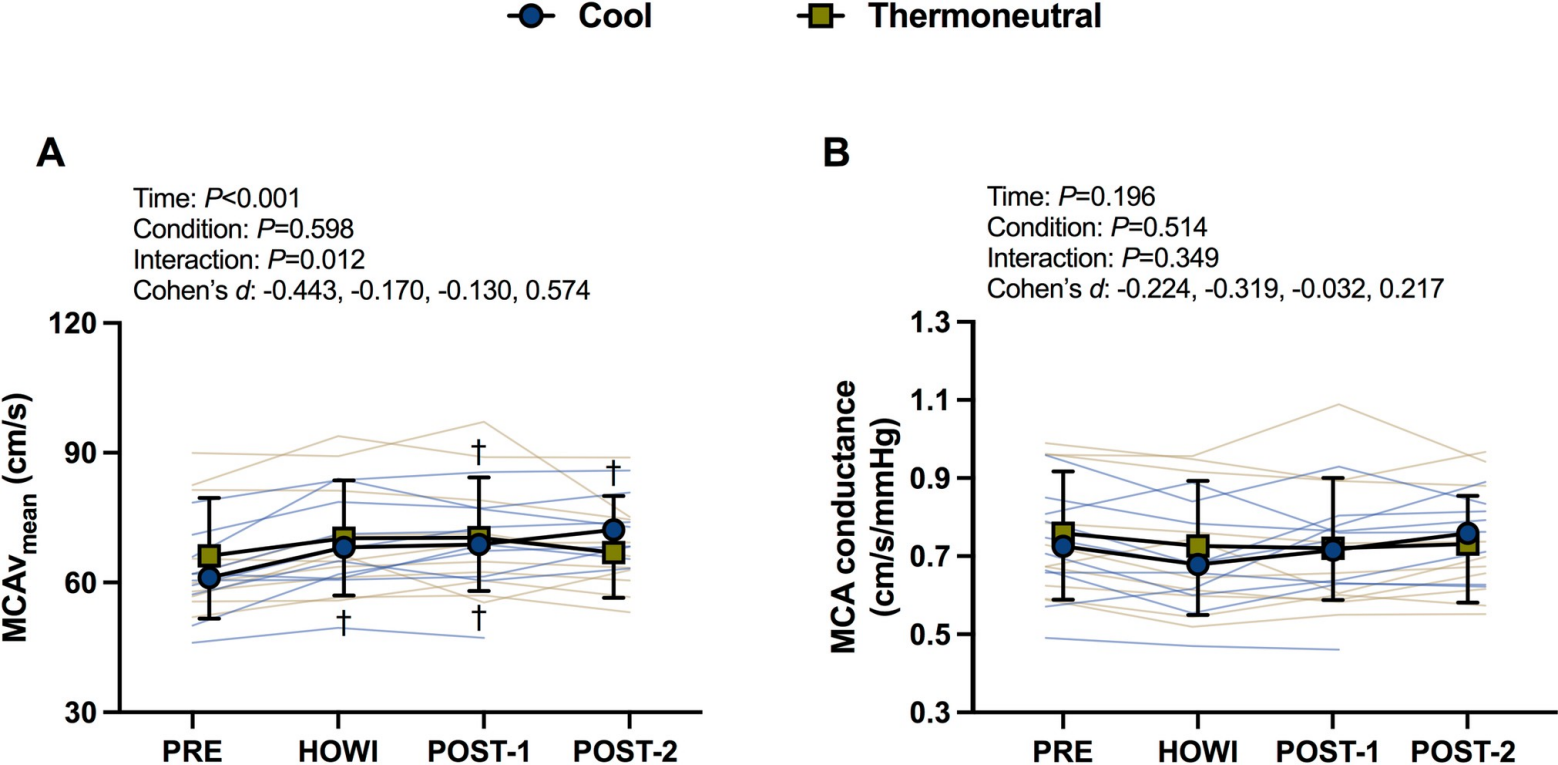
Cerebrovascular responses at resting baseline. MCA mean blood velocity (A) and MCA conductance (B) at baseline before immersion (PRE), 30 minutes of head-out water immersion (HOWI), immediately post-immersion (POST-1) and 45 minutes post-immersion (POST-2) in thermoneutral (35*°*C) and cool water (25*°*C). Linear mixed-effects models were used to compare the absolute mean values. *Post hoc* tests using the Bonferroni’s correction for multiple comparisons were used where appropriate. Cohen’s *d* values are presented as PRE, HOWI, POST-1, and POST-2. n = 10 at PRE, HOWI, and POST-1, and n = 8 at POST-2.

Cerebrovascular variables were not different between conditions at PRE (**Table 1**). In TN, MCAv_mean_ was not statistically different from PRE to HOWI (*P* = 0.063) but was greater than PRE at POST-1 (*P* = 0.050). In COOL, MCAv_mean_ was greater than PRE at every time point (all *P*<0.001). MCAv_mean_ was not different between conditions (all *P*≥0.320) (**Fig 4A**). MCA conductance was not different within or between conditions (**Fig 4B**).

#### Cerebrovascular reactivity to CO_2_ responses

**Fig 5:**
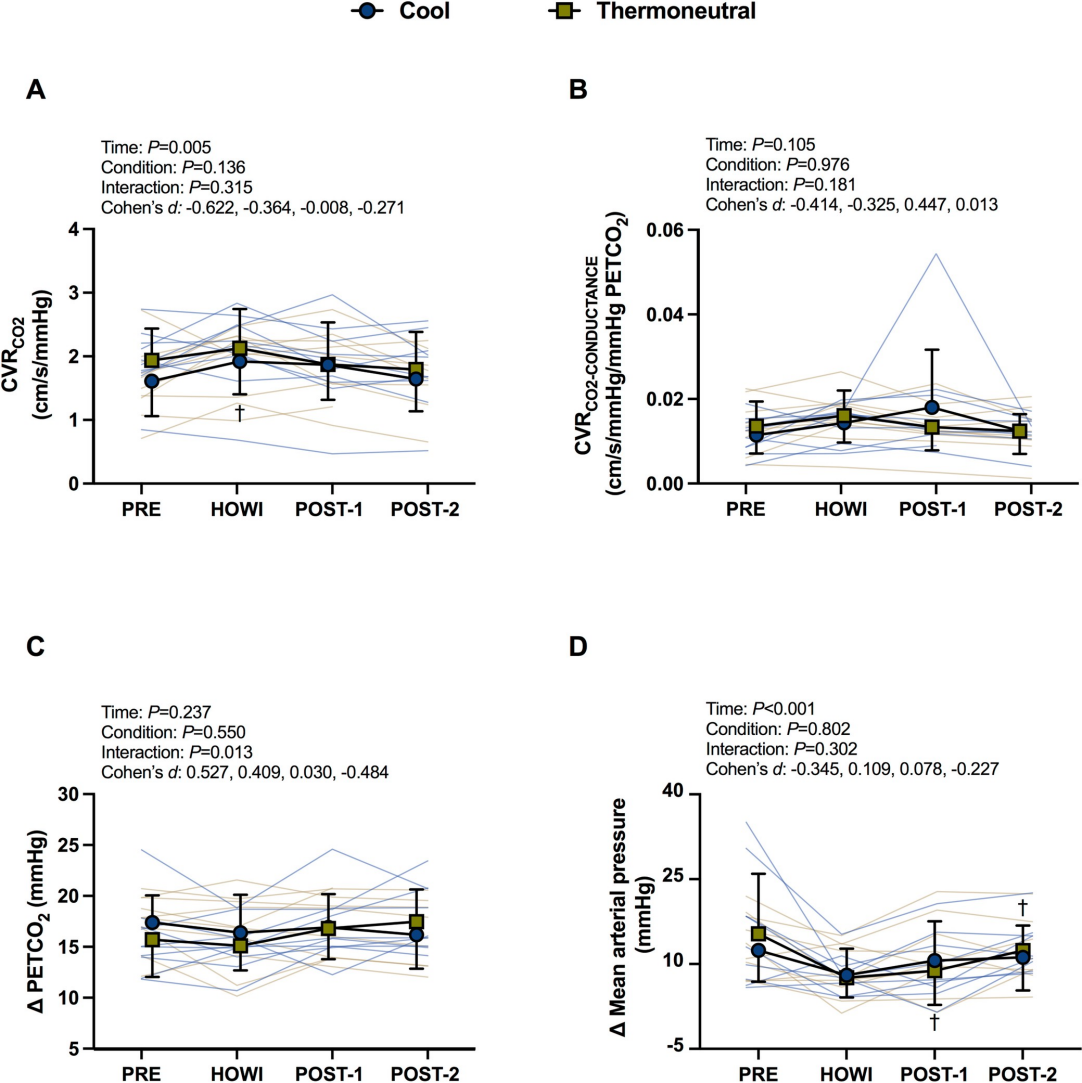
Cerebrovascular responses during hypercapnia. Cerebrovascular reactivity to CO_2_ for MCA mean blood velocity (A) and cerebrovascular reactivity to CO_2_ for MCA conductance (B) during the hypercapnic protocol at pre-immersion (PRE), 30 minutes of head-out water immersion (HOWI), immediately post-immersion (POST-1) and 45 minutes post-immersion (POST-2) in thermoneutral (35*°*C) and cool water (25*°*C). The change in end-tidal CO_2_ tension (C) and the change in mean arterial pressure (D) from baseline to the last 30 seconds of the hypercapnic protocol are calculated at pre-immersion (PRE), 30 minutes of head-out water immersion (HOWI), immediately post-immersion (POST-1) and 45 minutes post-immersion (POST-2) in thermoneutral (35*°*C) and cool water (25*°*C). Linear mixed-effects models were used to compare the absolute mean values. *Post hoc* tests using the Bonferroni’s correction for multiple comparisons were used where appropriate. † = different than PRE. * = different between conditions. Cohen’s *d* values are presented as PRE, HOWI, POST-1, and POST-2. CVR_CO2_ and CVR_CO2-CONDUCTANCE_: n = 10 at PRE, HOWI, and POST-1, and n = 8 at POST-2. Δ PETCO_2_: n = 10 at PRE, HOWI, POST-1, and n = 8 at POST-2. Δ MAP: n = 9 at PRE, HOWI, and POST-1, and n = 8 at POST-2.

CVR_CO2_ and CVR_CO2-CONDUCTANCE_ were not different between conditions at PRE (**Table 1**). The mixed-effects model revealed a significant time effect for absolute CVR_CO2_ (*P* = 0.005). In TN, CVR_CO2_ did not change from PRE (all *P*≥0.365). In COOL, CVR_CO2_ increased from PRE during HOWI (*P* = 0.040) (**Fig 5A**). CVR_CO2-CONDUCTANCE_ was not different within or between conditions (**Fig 5B**). The mixed-effects model revealed a significant interaction foe increase in PETCO_2_ during the hypercapnic protocol (*P* = 0.013) but no main effect of time (*P* = 0.237) or condition (*P* = 0.550). The hypercapnic protocol increased PETCO_2_ similarly in TN and COOL at PRE (16±4, 17±3 mmHg), HOWI (15±2,16±4 mmHg), POST-1 (17±3, 17±3 mmHg), and at POST-2 (18±3, 16±3 mmHg) (**Fig 5C**). The mixed-effects model revealed a significant time effect for the change in mean arterial pressure (*P*<0.001) but no condition effect (*P* = 0.802) (**Fig 5D**). In TN, the hypercapnic protocol increased mean arterial pressure more at PRE (12±6 mmHg) vs. HOWI (8±5 mmHg; *P*<0.001) and POST-1 (7±13; *P* = 0.003). In COOL, the increase in mean arterial pressure during stepped hypercapnia tended to be greater in PRE (15±11 mmHg) vs. HOWI (8±3 mmHg; *P* = 0.052).

### Common carotid artery hemodynamics

**Fig 6:**
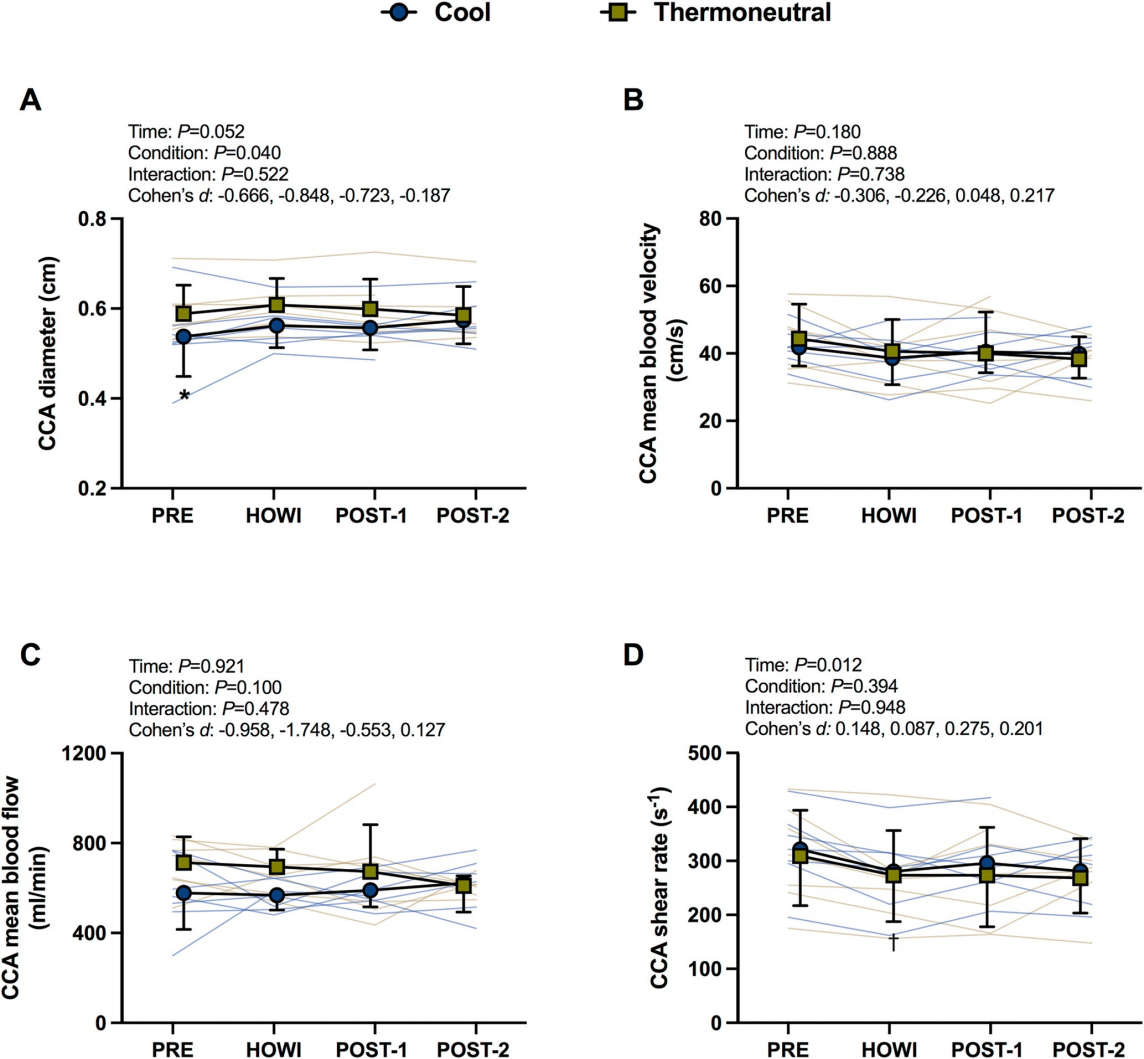
Common carotid artery responses. CCA diameter (A), CCA mean blood velocity (B), CCA mean blood flow (C), and CCA shear rate (D) at pre-immersion (PRE), 30 minutes of head-out water immersion (HOWI), immediately post-immersion (POST-1) and 45 minutes post-immersion (POST-2) in thermoneutral (35*°*C) and cool water (25*°*C). Linear mixed-effects models were used to compare the absolute mean values. *Post hoc* tests using the Bonferroni’s correction for multiple comparisons were used where appropriate. † = different than PRE. * = different between conditions. Cohen’s *d* values are presented as PRE, HOWI, POST-1, and POST-2. n = 7 at PRE, n = 6 at HOWI, n = 7 at POST-1, and n = 6 at POST-2.

CCA diameter was greater at PRE in TN (*P* = 0.050, *d* = -0.666). CCA diameter was not different over time in TN (all *P*≥0.161) and COOL (*P*≥0.082) (**Fig 6A**). There was no significant main effect or interaction for CCA mean blood velocity (**Fig 6B**) and mean blood flow (**Fig 6C**). CCA shear rate exhibited a main effect of time (*P* = 0.012). In TN, CCA shear rate was lower than PRE during HOWI (*P* = 0.046). In COOL, CCA shear rate tended be lower than PRE during HOWI (*P* = 0.066). CCA shear rate was not different between conditions (all *P*>0.999) (**Fig 6D**).

## Discussion

We investigated the acute effects of thermoneutral and cool HOWI on cerebrovascular function in healthy, young adults. Contrary to our hypotheses, we observed similar MCAv_mean_ responses in cool and thermoneutral water immersion. Despite an increase in MCAv_mean_ during and following cool water immersion, and immediately following thermoneutral water immersion, the increase(s) did not translate to a change in cerebrovascular reactivity to hypercapnia.

Cool and thermoneutral water immersion elicited similar MCAv_mean_ responses at 30 minutes of HOWI and immediately following HOWI that are likely contributed by modulators of cerebral blood flow. During and immediately following HOWI, the change in PETCO_2_ and mean arterial pressure were similar between cool and thermoneutral water immersion. However, the change in MCAv_mean_ was ~9 cm/s greater at 45 minutes post-cool HOWI compared to thermoneutral HOWI (15% vs. 2% increase from PRE). It is important to note that these responses observed at 45 minutes post-immersion had large inter-subject variability in both the cool (range: 5–37%) and thermoneutral (range: -16 to 11%) condition. Nonetheless, we speculate that the higher MCAv_mean_ at this timepoint (not statistically different) may partly be explained by PETCO_2_ and mean arterial pressure. First, PETCO_2_ was ~1–2 mmHg greater in the cool versus thermoneutral visit. Albeit PETCO_2_ was not statistically different within or between conditions, our participants exhibited a 1–5% increase in MCAv_mean_ per 1 mmHg increase in PETCO_2_, respectively. Therefore, PETCO_2_ may contribute to greater MCAv_mean_ at 45 minutes post-cool HOWI, but we did not measure tidal volume or ventilation, which may have allowed some insight into the non-statistical differences in PETCO_2_. Second, there was no condition effect for mean arterial pressure, but it was elevated at 45 minutes post-cool HOWI, at which point MCAv_mean_ also remained elevated in the cool condition. It is possible that higher mean arterial pressure contributed to an increase in MCAv_mean_ at 45 minutes post-cool HOWI, when MCAv_mean_ was no longer elevated in the thermoneutral condition. However, MCA conductance did not differ over time or between conditions. Thus, it may not be mean arterial pressure *per se* that is contributing to the changes in MCAv_mean_, but the ability of the cerebral arteries to buffer changes in pressure (i.e., cerebral autoregulation). We did not directly measure cerebral autoregulation, but others [40] have demonstrated that dynamic cerebral autoregulation is attenuated at 20 minutes of head-out immersion in 22°C water using bilateral thigh cuff deflation that rapidly decreases blood pressure. It is unclear if this reduction (or ‘worsening’) in dynamic cerebral autoregulation occurs during a hypertensive stimulus or if it persists during recovery from cool water immersion. To these points, static and dynamic cerebral autoregulation did not change during or following mild whole-body cold stress (using a water perfused suit) when mean arterial pressure was increased by 5±3 mmHg and 6±4 mmHg, respectively [41]. Therefore, we speculate that cerebral autoregulation was likely similar during and following cool water immersion. However, without a direct measure of cerebral autoregulation in our study, we cannot determine if this component of cerebrovascular function was altered during or following cool water immersion and if this potentially contributed to the MCAv_mean_ response at 45 minutes following cool HOWI.

There was substantial interindividual variability in the MCAv_mean_ response to thermoneutral HOWI. Although not statistically significant (P = 0.063), thermoneutral HOWI increased MCAv_mean_ by ~4 cm/s (range: -1 to 11 cm/s; Δ: 6±7%) compared to pre-HOWI, which is in agreement with our previous work [9]. The cerebral hemodynamic response to water immersion is likely modulated by changes in end-tidal CO_2_ tension and mean arterial pressure. During the thermoneutral visit, we observed no change in PETCO_2_ (1±2 mmHg) but an increase in mean arterial pressure (11±7 mmHg) at 30 minutes of head-out immersion in 35°C water. These findings differ slightly from prior work. Under comparable experimental conditions, we have previously observed a similar increase in PETCO_2_ (~1–2 mmHg), no change in mean arterial pressure, and a modest increase in MCAv_mean_ (~4 cm/s) at 30 minutes of thermoneutral HOWI [9]. Carter *et al*. [8] reported a similar increase in mean arterial pressure (~12 mmHg) but a greater increase in PETCO_2_ (~5 mmHg) and MCAv_mean_ (~12 cm/s) during 10 minutes of standing immersion in 30°C water. Although the reason(s) for the discrepancies between studies are yet to be elucidated, it could be due to body position (i.e., seated vs. standing), water temperature (30°C vs. 35°C), and/or immersion duration (10 minutes vs. 30 minutes).

Contrary to our hypothesis, cool HOWI increased MCAv_mean_ by 7±6 cm/s from dry baseline (range: -1 to 18 cm/s; Δ: 12±9%) and the increase was sustained immediately post- (Δ: 8±5 cm/s, 13±10%) and persisted to 45 minutes post-HOWI (Δ: 9±5 cm/s, 15±10%). Our MCAv_mean_ and mean arterial pressure responses to cool HOWI are similar to that of Carter *et al*. [8] in 30°C water; however, PETCO_2_ was augmented to a greater extent (~4 mmHg). Shoemaker *et al*. [12] also utilized a slightly warmer water temperature than ours (~28–30°C) and observed no significant change in PETCO_2_ and mean arterial pressure, but MCAv_mean_ increased by 12% during standing immersion. Thus, it appears that the cool water (~25°C) we employed and thermoneutral water (~28–35°C) can elicit similar increases in middle cerebral artery blood velocity while immersed.

Albeit cool HOWI augmented MCAv_mean_, it is unclear if blood flow in the internal carotid artery (ICA), that directly supplies the MCA, was altered. We chose to measure the CCA instead of the ICA as our extracranial vessel of interest since changes within this major artery are an indicator of cardiovascular and cerebrovascular health [20–22]. We did not observe a change in CCA blood flow during and following cool or thermoneutral HOWI, but we cannot assume a similar response occurred in the ICA. Rasch and Cabanac observed no change in facial skin blood flow during cold water immersion (~21.5°C up to the armpits for 10 minutes) compared to normothermia [42], thus, a redistribution of blood flow from the external carotid artery (ECA) to the ICA likely does not occur during cool water immersion. Gibbons *et al*. [43] previously reported a decrease in both ICA and ECA blood flow during cold stress, whereas others have reported no change in ICA blood flow [44]. These studies utilized a water-perfused suit, which does not provide the same stimulus (i.e., absence of hydrostatic pressure) as water immersion. Based on prior work, and no change in CCA blood flow (during or following cool HOWI) in the present study, we speculate that ICA blood flow was likely unaltered throughout the cool HOWI visit. However, we cannot confirm this and future investigations are warranted to determine if ICA blood flow is altered during cool water immersion.

We observed no change in cerebrovascular reactivity to CO_2_ during or following cool and thermoneutral HOWI. Thermoneutral HOWI did not alter cerebrovascular reactivity to CO_2_, which differs from our prior work, where cerebrovascular reactivity to CO_2_ decreased during and following 1 h of head-out immersion in 35°C water compared to pre-immersion baseline [9]. The reduction in our previous study may have been contributed to by mild elevations in PETCO_2_ (~2 mmHg) since we also observed lower cerebrovascular reactivity to CO_2_ during a dry condition while participants inhaled 13% CO_2_ (21% O_2_ and 66% N_2_) to match PETCO_2_ during HOWI [9]. In the present study, we did not observe a significant increase in PETCO_2_ (~1–2 mmHg) and we used a 9 minute fixed inspired stepped hypercapnia protocol versus a 3.5 minute rebreathe protocol in our previous work [9]. The CO_2_ duration and achievement of steady state likely contributed to the contradictory findings. Burley *et al*. [45] demonstrated differences in absolute and relative cerebrovascular reactivity during 5% CO_2_ inhalation of different durations (1, 2, 4, and 5 minutes). However, Carr *et al*. [46] reported similar cerebrovascular reactivity values at 2–5 minutes of dynamic end-tidal forcing (P_CO2_ +9.4 mm Hg) since steady state was achieved. In this regard, our present findings likely differ from our prior work since a steady state was not attained during the rebreathe test.

To our knowledge, we are the first to examine cerebrovascular reactivity to CO_2_ during and following an acute bout of cool HOWI. Our results indicate that cerebrovascular reactivity to CO_2_ is not altered during or following an acute bout of cool HOWI in healthy, young adults. These individuals presumably demonstrated a healthy cerebrovascular response to hypercapnia at rest and this response was not altered as a result of a single session of cool water immersion. It is important to note that these cerebrovascular results are specific to the middle cerebral artery during brief periods of hypercapnia only. It is unclear if cool water immersion alters reactivity in other intracranial arteries, in response to hypocapnia, or in diseased populations that may have altered cerebrovascular function at rest.

### Experimental considerations

Our study has several limitations that pertain to data interpretation. First, we had a relatively modest sample size (*n* = 10) which may limit the interpretation of our findings. In the interest of transparency, we included both the individual values (where possible) and Cohen’s d effect sizes to account for our small sample size. Second, we obtained images of the CCA for only 7 of the 10 participants due to scheduling conflicts with the sonographer (*n* = 2) and change in insonation angle throughout the cool water visit (*n* = 1). Third, we did not have a direct measure of intracranial artery blood flow. Transcranial Doppler ultrasound is a useful tool that allows us to non-invasively measure beat-to-beat changes in intracranial artery blood velocity [17]. Since this technique does not allow us to measure vessel diameter, we assume diameter remains constant and that blood velocity is reflective of blood flow. MRI data has demonstrated contradictory findings on the influence of hypercapnia on intracranial vessel diameters. Verbree *et al*. [47, 48] reported no statistical change in MCA diameter (+1.5%) from normocapnia to +7.5 mmHg, whereas Coverdale *et al*. [49] observed an 8% increase in MCA diameter when PETCO_2_ was 9 mmHg greater than baseline. We observed modest changes in PETCO_2_ from our baseline periods (range: -5 to 6 mmHg), thus it is unlikely that MCA diameter changed at rest. However, we cannot rule this out without a direct measure of MCA diameter, which is impractical during water immersion. Fourth, CO_2_ stimulus duration, data extraction, and measurement technique (e.g., TCD vs. MRI) can impact relative and absolute cerebrovascular reactivity data [45, 50]. Thus, caution is warranted when comparing and interpreting cerebrovascular reactivity results across studies that utilized different approaches. Fifth, we did not measure ventilatory sensitivity to CO_2_ during the hypercapnic challenge, which has previously been shown to alter the cerebral hemodynamic response to CO_2_ during hypo- and hyperventilation [51]. However, Howe *et al*. [52] recently demonstrated that changes in mean arterial pressure, not the magnitude of ventilation, can influence cerebrovascular reactivity to CO_2_. Our cerebrovascular reactivity results using MCA conductance demonstrate that the increase in blood pressure during stepped hypercapnia likely did not influence MCAv reactivity. Sixth, we did not assess cerebrovascular reactivity to hypocapnia, which could be relevant to immersion in much colder water temperatures that elicit the cold shock response. Seventh, we did not include a non-immersed time control visit to determine the direct effects of immersion and time on the cerebrovascular response. We utilized the thermoneutral water immersion visit as our control, to compare the effects of water immersion temperature on the cerebrovascular responses to head-out immersion. Lastly, women were tested during the first 10 days of menstruation when progesterone and estrogen are at their lowest due to the known vascular effects of menstrual cycle phase on cerebral hemodynamics [53, 54]. However, it is unknown if there is an interaction between menstrual cycle phase and the cerebrovascular response to water immersion.

### Perspectives and significance

These data indicate that cool water immersion increases MCAv_mean_ during and following immersion, which might translate to an increase in intracranial artery shear stress, assuming vessel diameter remains constant. The increase in MCAv_mean_ during cool water persisted 45 minutes following immersion whereas it returned to pre-immersion values in the thermoneutral condition. Thus, cool water provided a more prolonged stimulus for potential vascular improvements. This potential shear stimulus associated with the increase in MCAv_mean_ could increase nitric oxide levels in cerebral endothelial cells [1] and improve local vasodilator function (i.e., cerebrovascular reactivity to CO_2_). However, common carotid artery shear rate was reduced in both thermoneutral and cool HOWI. Since neither condition elicited a shear rate known to stimulate improvements in large conduit vessel function (i.e., common carotid artery), it is possible that these water temperatures and durations of immersion are not an effective intervention for improving vascular function in large extracranial arteries. We previously demonstrated that hot (39°C) HOWI augments CCA blood flow and shear rate likely due to increased blood flow to the external carotid artery [10] and internal carotid artery [55]. Therefore, head-out water immersion may be a beneficial tool for improving vascular health in extracranial arteries using hot water and in intracranial arteries using cool water. However, it is important to determine if episodic increases in intracranial artery blood velocity translate to beneficial vascular adaptations. Additional research aimed at exploring potential regional differences in cerebral hemodynamics following repetitive cool and hot HOWI is warranted. We aim to further explore the acute effects of other temperatures/durations of HOWI on cerebrovascular control to inform future interventions for improving intra- and extracranial arterial function.

## Conclusion

We observed no differences between middle cerebral artery blood velocities at 30 minutes of cool and thermoneutral head-out water immersion and immediately following 45 total minutes of water immersion. The increase in middle cerebral artery blood velocity persisted for 45 minutes following cool water immersion only. Despite the hydrostatic pressure of the water, neither cool or thermoneutral water promoted an increase in blood flow or shear rate through the common carotid artery. Finally, cerebrovascular reactivity to CO_2_ was not altered during or following cool and thermoneutral head-out water immersion. In sum, cool water immersion may provide a beneficial stimulus for vascular adaptations in the middle cerebral artery due to augmented blood velocity during and following immersion. These preliminary data can be utilized to inform potential future aquatic interventions for increasing intracranial artery blood velocity in humans.

## Supporting information

S1 Data (XLSX)
